# Association of Body Condition Score with Ultrasound Measurements of Backfat and Longissimus Dorsi Muscle Thickness in Periparturient Holstein Cows

**DOI:** 10.3390/ani11030818

**Published:** 2021-03-15

**Authors:** Nektarios Siachos, Georgios Oikonomou, Nikolaos Panousis, Georgios Banos, Georgios Arsenos, Georgios E. Valergakis

**Affiliations:** 1 Laboratory of Animal Husbandry, Faculty of Veterinary Medicine, School of Health Sciences, Aristotle University of Thessaloniki, Box 393, 54124 Thessaloniki, Greece; nsiachos@vet.auth.gr (N.S.); banos@vet.auth.gr (G.B.); arsenosg@vet.auth.gr (G.A.); 2 Department of Livestock and One Health, Institute of Infection, Veterinary & Ecological Sciences, Faculty of Health and Life Sciences, University of Liverpool, Neston CH64 7TE, UK; Georgios.Oikonomou@liverpool.ac.uk; 3 Clinic of Farm Animals, Faculty of Veterinary Medicine, School of Health Sciences, Aristotle University of Thessaloniki, 54124 Thessaloniki, Greece; panousis@vet.auth.gr; 4 Scotland’s Rural College and Roslin Institute, Edinburgh EH25 9RG, UK

**Keywords:** adipose, dairy cattle, longissimus dorsi, transition, ultrasonography

## Abstract

**Simple Summary:**

Association of body condition scoring with the amount and mobilization of backfat and skeletal muscle around calving in dairy cows, although essential, has not been sufficiently investigated yet. The metabolic state of subcutaneous fat can be safely predicted by repeated body condition scorings, but the metabolic state of muscle tissue must be assessed by repeated ultrasound measurements. The results enhance our on-farm ability to assess the nutrient reserves in dairy cows transitioning from gestation to lactation.

**Abstract:**

Most cows experience a period of nutrient deficit during the periparturient period. Body condition scoring (BCS) is widely used on farms to assess body nutrient reserves and mobilization. The aims of this study were to: (i) determine the association of BCS with ultrasound measurements of backfat (BFT) and longissimus dorsi muscle thickness (LDT) during the periparturient period of Holstein cows from different herds, accounting for potential sources of variation, such as herd, parity and period relative to calving and (ii) establish reference intervals (RIs) for BFT and LDT per BCS estimate. Two-hundred and fifty-two cows from six commercial farms were used. Body condition scores, BFT and LDT were assessed at seven time-points during the periparturient period. Assessments of BCS estimates as predictors of BFT and LDT and the contribution of BFT and LDT to BCS estimates were performed with the use of linear mixed models. Reference intervals for BFT and LDT per BCS estimate were established with the Reference Value Advisor. One unit of BCS change was associated with 8.2 mm of BFT and 10.9 mm of LDT pre- and postpartum. Range of BFT and LDT in established RIs per BCS was wide with significant overlap. Both subcutaneous fat and, to a lesser degree, skeletal muscle reserves contribute to BCS estimation. Repeated BCS estimations credibly predict energy balance status in periparturient dairy cows. The metabolic state of muscle tissue should be assessed by repeated ultrasound measurements.

## 1. Introduction

Dairy cows undergo significant metabolic and endocrinologic adaptations during the transition from late gestation to early lactation. Exponential fetal growth and initiation of lactation combined with the periparturient reduction in dry matter intake causes most cows the experience a period of negative energy balance [1]. This nutrient deficit results in mobilization of body energy and protein reserves. Dairy cows were found to mobilize 54 kg of adipose tissue and 21 kg of body protein during the first 5 weeks of lactation [2]. High yielding dairy cows mobilized up to 1 kg of tissue protein in milk per day during the first 7–10 days to provide amino acids, contributing to liver gluconeogenesis [3]. This nutrient deficit may well occur before calving [1].

Body weight (BW) is limited in predicting a cow’s nutrient reserves, being highly dependent on frame size, gut fill and uterus weight [4]. Moreover, changes in BW represent variable changes in the relative amount of body fat, protein or water [4].

Body condition score (BCS) is a widely used management tool for evaluating the proportional amount of fat in a cow or in a group of cows. It is a subjective method but the intra- and interevaluator agreement for BCS is relatively high [5,6,7,8]. Body condition score at calving, as well as BCS nadir and total BCS loss during early lactation, has been associated with milk production, reproductive performance and postparturient disease incidence [9], reflecting the importance of accurately monitoring the nutrient reserves and their mobilization during the periparturient period.

Several studies have investigated the validity of BCS estimates by measuring the ultrasound backfat thickness (BFT) alone [10,11,12] or in combination with longissimus dorsi muscle thickness (LDT) [13,14,15]. The latter, which is a combined approach, is more appropriate as both fat and muscle reserves can contribute to body condition scoring, which is a nontactile assessment method in dairy cows. However, only two studies focused at the periparturient period [14,15] for both BFT and LDT measurements and both included a rather small number of cows (72 and 91, respectively), all from the same herd, in each case. The relationship of BCS with BFT and LDT ultrasound measurements would ideally be investigated using a larger number of animals kept under various management conditions; such an approach would improve the study’s external validity and could further our understanding of these complex relationships.

Variation in BFT and LDT within each BCS estimate remains unclear. Schröder and Staufenbiel [4] reported, in an extended review, average expected BFTs per BCS estimate, without any information regarding measures of dispersion. Moreover, as reported data originate from an earlier publication [16], whether these values are valid for modern dairy cows is questionable. Furthermore, the expected LDT range per BCS estimate in Holstein cows has not been investigated yet. As a consequence, whether cows assigned the same BCS possess the same or similar energy and protein reserves remains unknown; neither the amount depleted nor deposited at the same BCS loss or gain, during a period of extensive tissue remodeling, can be quantified. Hence, the establishment of a reference range for BFT and LDT per BCS estimate is considered quite useful when studying fat and muscle mobilization in periparturient cows.

Therefore, the objective of this study was: (i) to assess BCS estimates as predictors of BFT and LDT and to quantify the simultaneous contribution of BFT and LDT to BCS during the dry period and early lactation in Holstein cows from different herds, accounting for various sources of variation, and (ii) to establish reference intervals for BFT and LDT, showing the range per BCS estimate.

## 2. Materials and Methods

### 2.1. Farms, Animals and Study Design

The study was conducted from September 2016 to October 2019. Two-hundred and fifty-two Holstein cows in different parities (1st: *n* = 14; 2nd: *n* = 101; 3rd: *n* = 72, and ≥4th: *n* = 65) from 6 commercial dairy farms (A: *n* = 32; B: *n* = 53; C: *n* = 20; D: *n* = 41; E: *n* = 51 and F: *n* = 55) were enrolled in this cohort study. Only purebred Holstein cows were included in the study. Farms kept 110 to 360 milking cows with an average milk yield of 9000 to 12,000 kg per cow per lactation. Multiparous cows had a mean (±SD) dry period duration of 64.1 (±27) days. Dry cows were housed in bedded packs, except for farm B and E where they were in free stalls. Fresh cows in all farms were housed in 2- or 3-row free-stall barns and were milked twice daily. Dry cows were fed total mixed rations (TMRs), delivered once daily, consisting mainly of corn silage, mixed cereal-legume silage, wheat straw and concentrates consisting mainly of corn, wheat brans, soybean meal and canola meal. Fresh cows were also fed TMRs, delivered once or twice daily, consisting mainly of corn silage, alfalfa hay, wheat straw and concentrates consisting mainly of corn, barley, triticale, sugar beet pulp, molasses, cottonseed, soybean meal and canola meal. Both dry and fresh cows also received appropriate amounts of macro- and microminerals and vitamins.

The body condition score (BCS), backfat thickness (BFT) and longissimus dorsi muscle thickness (LDT) of each cow was assessed at 7 time-points relative to the day of calving: −60d/−45d (for all but 1st parity cows; multiparous cows with a shorter dry period were assessed at drying-off); −21d; −7d; 0d; +7d; +21d and +28d (±2 days), by the first author, resulting in a total of 1659 records (Supplementary Materials). First parity cows were measured at −21d for the first time. Cows calving >5 days earlier than expected had no measurements for study day −7d.

Cows were minimally restrained with headlocks at the feed bunk. At first, cows were scored for BCS on a 5-point scale with 0.25-unit increments [6], in order to avoid biased estimation. Then, BFT and LDT were measured by real time B-mode ultrasonography, using a portable 5.0–7.5 MHz linear transducer (ImaGo S, IMV imaging, GB) at 80–100 mm depth. Examination sites were brushed to remove debris, but not clipped, and ultrasound gel was applied to couple the probe surface with the skin. Images were frozen and interpreted on-site by automatically measuring the distance between two points, set manually on the screen using specific landmarks, with an accuracy of 0.1 mm. For BFT measurements, the probe was placed lightly on the sacral area, vertically on an imaginary line connecting the pin (tuber ischii) and the hook (tuber coxa), at the point corresponding to the cranial end of the first coccygeal vertebra, as described by [4]. The BFT measurements always included the skin thickness and the profound fascia was used as a landmark to distinguish backfat from the gluteal muscle. For LDT measurements, the probe was placed perpendicularly to the vertebral column on the transverse process of the 4th lumbar vertebra, at the site of the larger diameter of the muscle between the fasciae corresponding to the lateral edge of the multifidus dorsi muscle, as described by [17].

### 2.2. Statistical Analysis

Pairwise correlations (r) for BCS, BFT and LDT were calculated for the dry period, the lactation and the whole study period.

Differences in BCS (Δ_BCS), BFT (Δ_BFT) and LDT (Δ_LDT) were calculated by subtracting the previous measurement from each measurement for the same cow. Both Δ_BFT and Δ_LDT were regressed against Δ_BCS in order to assess the rate of change in Δ_BCS and Δ_LDT per unit of Δ_BCS change.

An assessment of BCS estimates as predictors of BFT and LDT separately, considering the effects of period and parity, and adjusting for cow- and herd-level random variation by building a nested term, was performed with linear mixed effects models using time-points to specify within-subjects repeated observations (model 1):

Y_ij_ = μ + BCS + Period_i_ + Parity_j_ + Period_i_ × Parity_j_ + Cow(herd)_ij_ + e_ij_, (1)

where Y_ij_ = BFT or LDT, μ = overall mean, BCS = the fixed effect of BCS as covariate, Period_i_ = the fixed effect of the ith period (2 levels: dry period or lactation), Parity_j_ = the fixed effect of the jth parity (4 levels: 1st; 2nd; 3rd; ≥4th), Period_i_ × Parity_j_ = the fixed effect of the interaction of the ith period with the jth parity, Cow(herd)_ij_ = the random effect of each cow nested within each herd for the ith period and the jth parity, and e_ij_ = the residual error.

The simultaneous contribution of BFT and LDT to BCS estimates, considering the effects of period and parity, and adjusting for cow- and herd-level random variation by building a nested term, was assessed with linear mixed effects models using time-points to define within-subjects repeated observations (model 2):

Y_ij_ = μ + BFT + LDT + Period_i_ + Parity_j_ + Period_i_ × Parity_j_ + Period_i_ × BFT + Period_i_ × LDT + Parity_j_ × BFT + Parity_j_ × LDT + Cow(herd)_ij_ + e_ij_, (2)

where Y_ij_ = BCS estimate, μ = overall mean, BFT = the fixed effect of BFT as covariate, LDT = the fixed effect of LDT as covariate, Period_i_ = the fixed effect of the ith period (2 levels: dry period or lactation), Parity_j_ = the fixed effect of the jth parity (4 levels: 1st; 2nd; 3rd; ≥4th), Period_i_×Parity_j_ = the fixed effect of the interaction of the ith period with the jth parity, Period_i_×BFT = the fixed effect of the interaction of the ith period with BFT, Period_i_ × LDT = the fixed effect of the interaction of the ith period with LDT, Parity_j_ × BFT = the fixed effect of the interaction of the jth parity with BFT, Parity_j_ × LDT = the fixed effect of the interaction of the jth parity with LDT, Cow(herd)_ij_ = the random effect of each cow nested within each herd for the ith period and the jth parity, and e_ij_ = the residual error.

Several diagnostic tests (variance inflation factor (<10); condition index at the lowest eigenvalue row (<15) and variance proportion (<0.90)) were performed to assess possible collinearity between BFT and LDT when regressed against BCS. All tests precluded any collinearity problem in the model. Factors with nonsignificant effects at the *p* > 0.20 level at the initial screening were removed from the final models. Among the covariance structures assessed (diagonal, compound symmetry, first-order autoregressive structure with homogenous (AR1) and heterogenous (ARH1) variances), the ARH1 covariance matrix yielded the best fit, resulting in the lower Akaike’s information criterion value. Assumptions of normality and homoscedasticity for the linear models were assessed with the visual observation of the Q-Q and predicted values vs. residuals plots, respectively. At significant F values for factors with >2 levels, pairwise comparisons between the estimated marginal means were performed using the Bonferroni confidence interval adjustment.

All analyses were performed with IBM SPSS v.25 (Armonk, NY, USA: IBM Corp.). Significance level was set at *p* < 0.05.

The predicted BFT and LDT values produced from model 1, adjusted for all other fixed and random effects fitted in the model, were used to calculate reference intervals (RIs) for BFT and LDT per BCS estimate, separately, with the Reference Value Advisor (v.2.1) software (RefValAdv), a set of macroinstructions developed for Microsoft Excel [18]. The RefValAdv report displays descriptive statistics, normality tests (Anderson–Darling test with histograms and Q-Q plots), outlier tests (Dixon’s and Tukey’s tests) and calculates 95% RIs with 90% confidence intervals (CIs), using parametric, Box-Cox transformation or nonparametric methods, according to normality and symmetry of data distribution, outliers and sample size. Moreover, regression-based RIs were also established for BFT and LDT with BCS as covariate. For this method, RefValAdv calculates 95% regression-based RIs with 90% CI for parametric and nonparametric models; in our case, parametric models assessed linear (untransformed or after Box-Cox transformation) and polynomial relationships—either homoscedastic or heteroscedastic. The RefValAdv output for parametric models displays the multiple R^2^ and the *p*-value of the model, regression and analysis of variance tables, a plot with regression lines for the fitted value and the upper and lower RI limits and residuals’ plots. Records regarding BCS < 2.25 and BCS > 4.00 were not enough to calculate RIs for these scores from our data, with any of the methods used.

## 3. Results

Descriptive data for BCS, BFT and LDT measurements at each time-point of the study are presented in Table 1. On average, cows moderately gained BCS, BFT and LDT from −60/−45 days to −7 days relative to the expected day of calving. Thereafter, they lost on average 0.54-unit BCS, 5.0 mm BFT (27.8%) and 9.5 mm LDT (25.4%) up to 28 days postpartum. Mean within-herd BFT and LDT loss during the same period ranged from 15.7 to 32.6% and from 19.0 to 33.3%, respectively.

All pairwise linear correlations are presented in Table 2. Backfat thickness had a high positive correlation with BCS (r = 0.839–0.867, *p* < 0.001); longissimus thickness had a moderate positive correlation with BCS (r = 0.688–0.722, *p* < 0.001). Backfat thickness and LDT were also moderately correlated (r = 0.639–0.696, *p* < 0.001).

**Figure 1:**
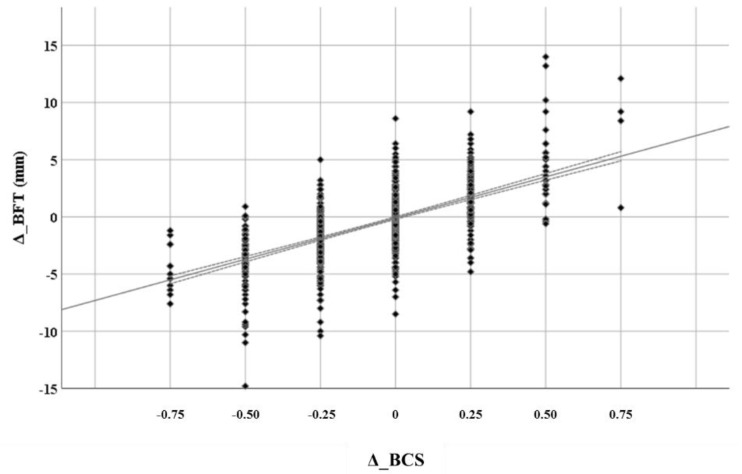
Rate of backfat thickness changes (Δ_BFT) across consecutive BCS changes (Δ_BCS) (r^2^ = 0.42, *p* < 0.001).

**Figure 2:**
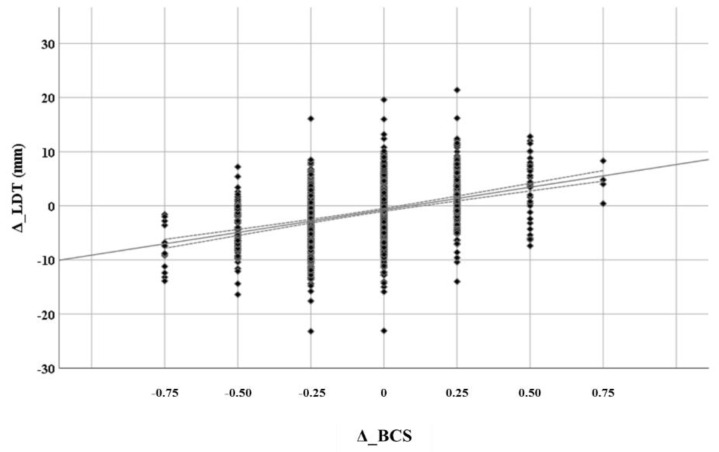
Rate of longissimus dorsi thickness changes (Δ_LDT) across consecutive BCS changes (Δ_BCS) (r^2^ = 0.14, *p* < 0.001).

The relationships of Δ_BFT and Δ_LDT with Δ_BCS in the same cow are depicted in Figure 1 and Figure 2. Both Δ_BFT and Δ_LDT had linear relationships with Δ_BCS. The slopes of Δ_BFT and Δ_LDT regressed against Δ_BCS were 7.2 (r^2^ = 0.42, *p* < 0.001) and 8.4 (r^2^ = 0.14, *p* < 0.001), respectively. Variability per Δ_BCS change was higher for Δ_LDT compared to Δ_BFT.

Regression coefficients for the association of BCS estimates as predictors of BFT and LDT are shown in Table 3. Each BCS-unit change was associated with 8.19 mm (SE = 0.182, *p* < 0.001) BFT and 10.88 mm (SE = 0.396, *p* < 0.001) LDT. The effects of period and period × parity interaction on BFT were nonsignificant (*p* = 0.155 and 0.133, respectively), but that of parity was significant (*p* < 0.001) (Table 4). Primiparous cows had lower BFT compared to that of multiparous cows (estimated marginal means 11.84 mm vs. 13.62–14.94 mm, *p* < 0.01). The effects of period and parity on LDT were significant (*p* < 0.001) (Table 5), but that of period×parity interaction was not significant (*p* = 0.108). Longissimus thickness during the dry period was higher than during lactation (estimated marginal means 31.75 mm vs. 29.01 mm, *p* < 0.001). Primiparous cows had lower LDT compared to that of multiparous ones (estimated marginal means 26.89 mm vs. 31.23–31.95 mm, *p* < 0.01).

Regarding the model assessing the contribution of BFT and LDT to BCS, in a descending F value order, BFT, LDT, period, period × LDT, parity and period × parity had a significant effect. The effects of period × BFT and parity × BFT were nonsignificant (*p* = 0.596 and *p* = 0.948, respectively) and were excluded from the final model. Parameter estimates for BFT and LDT were 0.057 (SE = 0.002, *p* < 0.001) and 0.012 (SE = 0.001, *p* < 0.001); LDT estimate for the dry period was reduced by 0.004 (SE = 0.001, *p* < 0.001) (Table 6).

According to the estimates produced, the contribution of BFT was 4.75 and 7.125 times (quotients from dividing the estimates produced for BFT by those for LDT) higher than that of LDT to BCS estimates during lactation and the dry period, respectively. Body condition scores during the dry period were higher than during lactation (estimated marginal means, 3.177 vs. 3.122, *p* < 0.001) (Table 7). Primiparous cows had higher BCSs than multiparous ones (estimated marginal means, 3.296 vs. 3.082, *p* < 0.01); similar differences were detected both during the dry period and lactation (Table 7).

The 95% RI with 90% confidence intervals of BFT and LDT measurements, adjusted for the effect of period relative to calving, parity and the random effect of each cow nested within each herd, calculated separately per BCS estimate for scores ranging from 2.25 to 4.00 are presented in Table 8. The RI within each BCS estimate, in most cases, was large, with the upper limit being approximately 50–100% higher than the lower one, both in BFT and LDT measurements.

**Figure 3:**
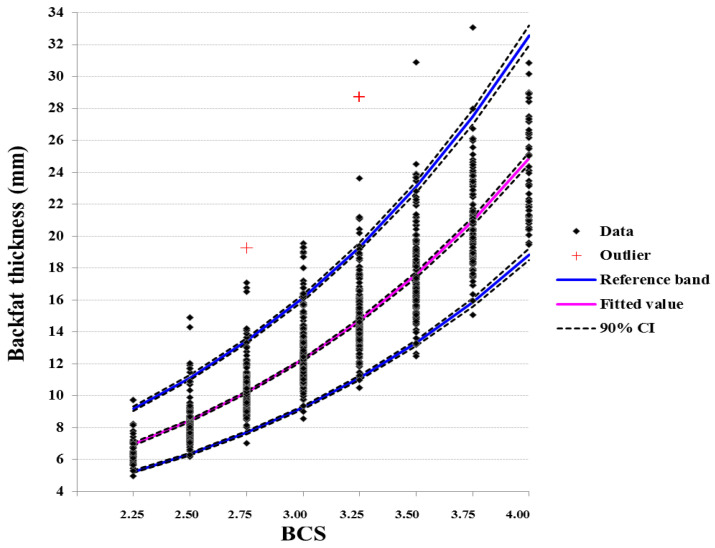
Regression lines of backfat thickness (BFT), adjusted for the effects of parity and period relative to calving and the random variation of each cow nested within each herd, after a Box-Cox transformation, showing 95% reference interval and fitted values per BCS estimate (R^2^ = 0.895, *p* < 0.001).

**Figure 4:**
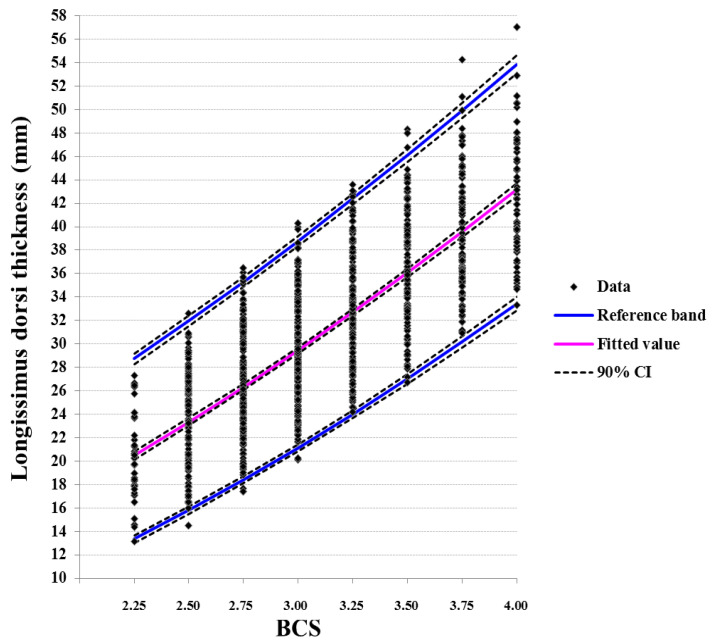
Regression lines of longissimus dorsi thickness (LDT), adjusted for the effects of parity and period relative to calving and the random variation of each cow nested within each herd, after a Box-Cox transformation, showing 95% reference intervals and fitted values per BCS estimate (R^2^ = 0.621, *p* < 0.001).

Moreover, the regression-based RI of the adjusted BFT and LDT measurements against BCS are depicted in Figure 3 and Figure 4. The parametric models after a Box-Cox transformation produced the best fit, yielding the higher multiple R2, for both BFT and LDT. Regression models explained 89.5 and 62.1% of the total dispersion for BFT and LDT RI, respectively. Upper and lower reference limits were quite similar to those produced by calculating RI separately for each BCS.

Despite the evident ascending order of these intervals at increasing BCS estimates, there was a notable overlap between successive scores. The overlap almost disappeared at 0.75-unit increments of BCS for BFT and at 1.25-unit increments of BCS for LDT.

## 4. Discussion

In this study, we determined the association of BCS with BFT and LDT measurements in periparturient Holstein cows, at six different farms, accounting for the effects of period relative to calving and parity. We also established RIs for BFT and LDT using the adjusted cow- and herd-level variation values, per BCS estimate. All assessments and measurements were performed by the same author, as suggested by [8].

Body condition score assesses the amount of adipose tissue in a cow with an acceptable accuracy [4]. Its validity has been confirmed via ultrasound BFT measurements. The correlation coefficient of ultrasound BFT with carcass measurements of backfat was r = 0.92 [19]. Moreover, ultrasound BFT measurements have a high repeatability; correlation coefficient between consecutive measurements was r = 0.975 [18].

We also found a high positive correlation between BCS and BFT. This is in agreement with other studies either small (44 periparturient multiparous Holstein cows) [12] or large-scale ones (1123 cows across different production stages) [11]. We also found a moderate correlation between BCS and LDT, in agreement with [20] (373 Holstein cows at early and late lactation).

Both BFT and LDT changed with quite a similar rate across BCS changes, with Δ_LDT showing a higher dispersion per Δ_BCS compared to Δ_BFT. This suggests a higher misclassification rate (values < 0 or >0 for positive or negative Δ_BCS, respectively) of muscle metabolic state, as measured by ultrasonography, according to changes between successive BCS estimations compared to that of fat.

Body condition score was a significant predictor of both BFT and LDT. This association was corrected for the period relative to calving and parity effects and for the random variation of each cow nested within each herd. Thickness of backfat and longissimus dorsi (8.19 and 10.88 mm, respectively) associated with 1-unit BCS change in this study differed substantially from those reported by [14]. However, in the latter study, reported BFT values were inexplicably low (range: 0.0–7.8 mm), especially considering that skin was included in BFT measurements; differences in BFT and LDT may be due to a different BCS scale used (6-point) and to lower overall BCS of cows used in the latter study. Nevertheless, in both studies, the LDT estimate associated with 1-unit BCS change was larger compared to that for BFT.

Our results confirmed previous work showing a significant contribution not only of adipose tissue, but also of skeletal muscle to BCS estimation in dairy cows during the periparturient period [14,15]. However, these studies suggest a higher contribution of LDT on BCS changes (0.050- and 0.048-unit per mm, respectively) than that of BFT (0.027- and 0.024-unit per mm, respectively) in transition cows. The opposite was the case in our study, where the mixed model analysis indicated that the contribution of each mm of BFT was 4.75 and 7.125 times higher than that of each mm of LDT on BCS, during lactation and the dry period, respectively. This discrepancy may be attributed to differences in the proportional amounts of fat and muscle mobilized in cows in each study. For instance, cows in the [15] study mobilized 45–50% of BFT and 15–20% of LDT, while in our study cows mobilized 28% of BFT and 25% of LDT. Differences in tissue mobilization may have resulted from differences in nutritional management; the variability in our study, which included 6 different herds, was much larger than that of previous studies that each included only one herd. Moreover, the authors of [15] aggregated the contribution of three different sites for skeletal muscle depth in the reported 0.048 estimate; the estimate for loin longissimus dorsi thickness alone was 0.013, about half that of BFT and similar to ours (0.012). Our results are in accordance with those of [13], which reported higher estimates for subcutaneous fat at the lumbar and pelvic areas compared to LDT, in a mixed model from 1271 observations in lactating Holsteins, Jerseys and Holstein × Jersey cows in four different feeding groups.

Jaurena et al. [14] reported an estimated marginal mean difference of 0.14-unit BCS between pre- and postcalving measurements across the same BFT and LDT values. Despite the similar significant effect of period relative to calving on BCS in our study, the estimated marginal mean difference between the dry period and early lactation was quite small (0.055-unit), indicating a negligible biological significance.

First, parity cows were assigned a higher BCS compared to multiparous ones at the same BFT and LDT. This is most likely due to differences in frame size. However, since the number of first parity cows in our study was relatively small, further research is needed to define whether this is actually true or a random finding.

Reference intervals of BFT and LDT per BCS estimate have not been reported in the literature. Schröder and Staufenbiel [16], as reported by [4], presented average values of BFT for each BCS. These values are in most cases higher than the upper limits of the RI in our study, confirming our hypothesis that average values produced more than two decades ago should be updated and accompanied by measures of dispersion. The established RI include a wide range of BFT and LDT measurements per BCS with significant overlap between successive scores, more notably on LDT. Regression models explained approximately 90 and 62% of the total dispersion for BFT and LDT RI, respectively, per BCS estimate, and produced similar upper and lower reference limits as when RIs were calculated separately per score. Therefore, RI established by either method can be used for interpretation of BCS records in periparturient Holstein cows.

The overlap of the BFT and LDT RIs between successive BCS, was high—on several occasions, this was > 90%. Overlap decreased considerably for 0.50-unit and 1.00-unit of BCS increments for BFT and LDT, while it almost disappeared at 0.75-unit for BFT and at 1.25-unit of BCS increments for LDT. In this respect, changes of 0.25-unit BCS cannot be safely accounted as actual differences in adipose tissue depots; this can be supported at 0.50-unit changes. Accordingly, actual differences in muscle tissue reserves can be safely accounted at 1.00-unit BCS changes, which, however, is of limited practical value.

Although the effects of body fat mobilization on health and productivity have been thoroughly studied in periparturient cows, similar research on muscle mobilization is missing. Body condition scoring is a strong predictor of adipose tissue reserves. On the other hand, as BCS is moderately associated with skeletal muscle reserves, at present, ultrasound measurements of LDT appear to be the only reliable method to quantify the degree and duration of muscle mobilization. In any case, we have the opinion that fat and skeletal muscle mobilization should be interpreted as concurrent metabolic adaptations from late gestation to early lactation but should be measured separately.

One limitation of this study was that the author who performed the BCS estimations also obtained the BFT and LDT measurements, which, although the former always preceded the latter, could have introduced a level of unconscious bias. However, the fact that all assessments were performed by the same evaluator, as is commonly performed in practice, warrants precision of the obtained measurements. Moreover, since farms were enrolled in the study consecutively and measurements were taken on one farm at a time, any season effects could not be distinguished from farm effects and therefore were not considered in our analysis. On the other hand, the main strength of this study was the inclusion of cows from several herds, extending the research over a broader range of biological variation compared to previous studies.

## 5. Conclusions

Body condition score is a strong predictor of subcutaneous fat reserves, but, to a lesser degree, of skeletal muscle reserves, in periparturient dairy cows. The main contributor to BCS estimates is BFT. Large overlapping of BFT and LDT RIs per BCS reveals significant variation of nutrient reserves in cows assigned the same score. Successive BCS estimations should be used on identifying cows losing or gaining subcutaneous fat but, regarding the metabolic state of muscle tissue, ultrasound measurements appear a more reliable alternative.

## Figures and Tables

**Table 1:** Descriptives for body condition score (BCS: 1–5 scale in 0.25 increments), backfat thickness (BFT) and longissimus dorsi muscle thickness (LDT) in 252 Holstein cows of 6 herds, from 60/45 days prepartum to 28 days postpartum.

		BCS				BFT (mm)				LDT (mm)			
Time-Point	n *	mean	SD	min	max	mean	SD	min	max	mean	SD	min	max
−60d/−45	237	3.21	0.53	2.25	4.50	15.04	6.89	5.20	41.00	34.00	9.72	14.80	63.00
−21d	249	3.29	0.46	2.25	4.50	15.93	6.30	4.80	44.80	35.19	8.31	16.40	61.30
−7d	218	3.34	0.47	2.25	4.50	16.58	6.52	5.20	47.20	35.47	9.19	14.40	69.80
0d	242	3.18	0.43	2.25	4.25	14.94	5.88	5.20	43.10	31.95	7.99	14.00	54.00
7d	241	3.06	0.43	2.00	4.25	13.90	5.70	5.20	32.80	29.87	8.26	12.80	61.70
21d	235	2.85	0.41	2.00	4.00	11.88	4.77	5.20	32.30	26.22	7.28	12.80	49.80
28d	237	2.79	0.42	2.00	4.00	11.34	4.46	4.80	34.80	25.93	7.24	12.40	48.80

*: number of records.

**Table 2:** Pearson’s correlation coefficients (r) for body condition score (BCS: 1–5 scale in 0.25 increments), backfat thickness (BFT) and longissimus dorsi muscle thickness (LDT) during the whole study period, the dry period and lactation in 252 Holstein cows of 6 herds.

**Overall**			
*n* = 1659	**BCS**	**BFT**	**LDT**
BCS	1	0.859 *	0.722 *
BFT		1	0.688 *
LDT			1
**Dry period**			
*n* = 704	**BCS**	**BFT**	**LDT**
BCS	1	0.867 *	0.691 *
BFT		1	0.696 *
LDT			1
**Lactation**			
*n* = 955	**BCS**	**BFT**	**LDT**
BCS	1	0.839 *	0.688 *
BFT		1	0.639 *
LDT			1

*n*: number of observations; * Correlation is significant at the 0.01 level.

**Table 3:** Linear mixed models showing the association of 1659 BCS records (1–5 scale in 0.25 increments) with backfat thickness (BFT) and longissimus dorsi thickness (LDT), adjusted for parity, period relative to calving and for the random variation of each cow nested within each herd, in 252 Holstein cows of 6 herds, from 60/45 days prepartum to 28 days postpartum.

Coefficient	Estimated Value	SE	*p*-Value	95% CI
BFT (mm)				
Intercept	−10.900	0.627	<0.001	−12.131–−9.669
BCS	8.189	0.182	<0.001	7.830–8.547
LDT (mm)				
Intercept	−3.660	1.316	<0.001	−6.242–−1.077
BCS	10.880	0.396	<0.001	10.104–11.656

**Table 4:** Estimated marginal means for backfat thickness (BFT) produced from a linear mixed model assessing the association of BCS with BFT, from 1659 records in 252 Holstein cows of 6 herds, from 60/45 days prepartum to 28 days postpartum, showing the effects of parity and period relative to calving, adjusted for the random variation of each cow nested within each herd.

	BFT (mm)	SE	*p*-Value	95% CI
Period			0.155	
Dry period	13.839	0.237		13.373–14.305
Lactation	13.361	0.228		13.212–14.110
Parity			<0.001	
1st	11.839 ^a^	0.718		10.424–14.142
2nd	13.616 ^b^	0.267		13.090–14.142
3rd	14.944 ^c^	0.316		14.322–15.566
+4th	14.602 ^c^	0.333		13.945–15.558

^a–c^ Different superscripts within the same column denote significant difference at the 0.05 level; estimated marginal means are adjusted for BCS = 3.103.

**Table 5:** Estimated marginal means for longissimus dorsi thickness (LDT) produced from a linear mixed model assessing the association of BCS with BFT, from 1659 records in 252 Holstein cows of 6 herds, from 60/45 days prepartum to 28 days postpartum, showing the effects of parity and period relative to calving, adjusted for the random variation of each cow nested within each herd.

	LDT (mm)	SE	*p*-Value	95% CI
Period			<0.001	
Dry period	31.754 ^a^	0.442		30.885−36.624
Lactation	29.014 ^b^	0.422		28.183−29.844
Parity			<0.001	
1st	26.887 ^a^	1.319		24.288−29.486
2nd	31.946 ^b^	0.490		30.981−32.912
3rd	31.228 ^b^	0.581		30.084−32.372
+4th	31.475 ^b^	0.611		30.271−32.679

^a–b^ Different superscripts within the same column denote significant difference at the 0.05 level; estimated marginal means are adjusted for BCS = 3.103 as covariate.

**Table 6:** Linear mixed model showing the contribution of backfat thickness (BFT) and longissimus dorsi thickness (LDT), adjusted for parity, relative to calving and the random variation of each cow nested within each herd, to 1659 BCS records (1–5 scale in 0.25 increments) in 252 Holstein cows of 6 herds, from 60/45 days prepartum to 28 days postpartum.

Coefficient	Estimated Value	SE	*p*-Value	95% CI
Intercept	1.831	0.036	<0.001	1.760–1.900
BFT (mm)	0.057	0.002	<0.001	0.054–0.060
LDT (mm)	0.012	0.001	<0.001	0.010–0.014
Dry period × LDT (mm) *	−0.004	0.001	<0.001	−0.006–−0.002

* Coefficient for Lactation × LDT (mm) was set as reference category for period × LDT effects (estimated value = 0); estimated marginal means for the fixed effects are adjusted for BFT = 14.22 mm and LDT = 31.22 mm as covariates.

**Table 7:** Estimated marginal means for BCS produced from a linear mixed model assessing the contribution of backfat thickness (BFT) and longissimus dorsi thickness (LDT) on BCS, from 1659 records in 252 Holstein cows of 6 herds, from 60/45 days prepartum to 28 days postpartum, showing the effects of parity and period relative to calving, adjusted for the random variation within each cow nested within each herd.

	BCS	SE	*p*-Value	95% Confidence Interval
Period			<0.001	
Dry period	3.177 ^a^	0.017		3.143–3.211
Lactation	3.122 ^b^	0.016		3.090–3.154
Parity			<0.001	
1st	3.296 ^a^	0.049		3.199–3.393
2nd	3.118 ^b^	0.018		3.082–3.154
3rd	3.102 ^b^	0.022		3.059–3.144
+4th	3.082 ^b^	0.023		3.038–3.127
Parity × Period			0.013	
Dry period				
1st	3.280 ^a^	0.055		3.171–3.389
2nd	3.146 ^b^	0.020		3.107–3.185
3rd	3.149 ^b^	0.024		3.102–3.196
+4th	3.132 ^b^	0.025		3.083–3.181
Lactation				
1st	3.312 ^a^	0.052		3.209–3.414
2nd	3.090 ^b^	0.019		3.052–3.128
3rd	3.054 ^b^	0.023		3.009–3.099
+4th	3.033 ^b^	0.024		2.985–3.080

^a–b^ Different superscripts within the same column denote significant differences at the 0.05 level; estimated marginal means are adjusted for BFT = 14.22 mm and LDT = 31.22 mm, as covariates.

**Table 8:** Descriptive statistics and reference intervals (RIs) for backfat thickness (BFT) and longissimus dorsi muscle thickness (LDT) per body condition score (BCS), adjusted for the effects of parity and period relative to calving and for the random variation of each cow nested within each herd, in 252 Holstein cows of 6 herds, from 60/45 days prepartum to 28 days postpartum.

			Descriptive Statistics					95% RI				
BCS	Item (mm)	*n*	mean	SD	median	min	max	Lower Limit and CI (90%)		Upper Limit and CI (90%)		Method
2.25	BFT	65	6.6	1.1	6.4	4.8	10.5	5.3	5.1–5.5	8.8	8.2–9.6	BCTSD
	LDT		20.6	5.1	20.0	12.8	34.4	13.7	12.8–14.6	27.9	26.3–29.6	BCTSD
2.50	BFT	188	8.3	1.8	7.7	5.6	15.6	6.7	6.2–7.0	12.0	10.9–14.9	NP
	LDT		23.3	5.3	22.8	12.4	37.2	16.0	14.5–16.5	30.6	29.7–32.6	NP
2.75	BFT	258	10.1	2.1	9.6	6.8	21.7	8.5	8.0–8.7	14.1	13.3–16.8	NP
	LDT		27.0	6.2	26.6	13.6	42.5	18.9	17.4–19.4	35.7	35.1–36.1	NP
3.00	BFT	406	12.5	2.7	12.1	7.0	22.5	10.0	9.8–10.3	18.6	17.2–19.0	NP
	LDT		29.1	5.9	28.8	15.6	44.5	22.3	21.9–22.7	38.2	37.1–38.5	NP
3.25	BFT	237	15.2	3.0	15.0	8.8	31.0	11.6	11.4–11.8	20.7	19.9–21.4	BCTSD
	LDT		33.1	6.6	32.8	19.2	56.0	24.6	24.2–25.5	42.5	41.4–43.1	NP
3.50	BFT	206	18.0	3.5	17.8	9.6	32.3	13.3	12.6–14.0	23.9	24.5–24.5	NP
	LDT		36.3	6.4	36.5	22.4	50.8	27.9	26.7–28.7	46.4	44.2–48.0	NP
3.75	BFT	148	20.9	3.4	20.3	15.0	32.1	16.7	15.1–17.4	26.3	25.6–33.1	NP
	LDT		39.6	7.2	38.6	24.4	61.7	31.1	30.9–31.9	50.2	47.8–54.3	NP
4.00	BFT	95	24.9	4.8	23.2	17.9	36.9	19.6	19.4–20.2	30.6	29.0–30.9	NP
	LDT		43.7	7.7	44.0	28.4	69.8	33.7	32.6–34.7	54.5	52.4–56.7	BCTSD

*n*: number of records; CI: confidence interval; BCTSD: Box-Cox transformed standard data; NP: nonparametric.

## Data Availability

The data presented in this study are available in the Supplementary Material.

## Supplementary Materials

The following are available online at https://www.mdpi.com/2076-2615/11/3/818/s1.
